# Association of Blood and Seminal Plasma Cadmium and Lead Levels With Semen Quality in Non-Occupationally Exposed Infertile Men in Abakaliki, South East Nigeria 

**Published:** 2017-06

**Authors:** Ademola C. Famurewa, Emmanuel I. Ugwuja

**Affiliations:** 1 Department of Medical Biochemistry, Federal University, Ndufu-Alike, Ikwo, Ebonyi State, Nigeria; 2 Department of Biochemistry, Ebonyi State University, Abakaliki, Nigeria

**Keywords:** Lead, Cadmium, Infertility, Semen Quality, Sperm Count, Reproductive Toxicity

## Abstract

**Objective:** To evaluate association of blood and seminal plasma lead and cadmium with sperm quality of non-occupationally exposed male partners of couples with infertility.

**Materials and methods:** A cross-sectional study was conducted on 75 men aged 20-45 years (mean = 37.1 ± 7.0 yrs.) with infertility recruited from the Fertility Clinic of a hospital in Abakaliki. Sperm count done in accordance with the WHO guidelines was used to classify the participants as normospamia, oligospermia and azospermia**. **Atomic absorption spectrophotometer was used to determine lead and cadmium levels in plasma from blood and semen.

**Results:** There were 15 azospermics, 22 oligospermics and 36 normospermics. Seminal and blood plasma cadmium as well as blood plasma lead were significantly (p < 0.01) higher in azospermic and oligospermic men compared to normospermic men. However, while seminal plasma lead was significantly (p < 0.05) higher in oligospermic and normospernic men than in azospermic men, the seminal plasma lead was comparable between oligospermic and normospermic men. Significant inverse associations (p < 0.01) were found between blood and seminal cadmium levels and sperm count, motility and morphology; blood lead was inversely correlated with sperm count only.

**Conclusion:** The study suggests that environmental exposure to cadmium and lead may contribute to development of poor sperm quality and infertility in men of reproductive age in Nigeria.

## Introduction

Infertility presents a worldwide reproductive health problem and its etiologic cause remains elusive. According to the WHO, infertility is the inability of a sexually active couple to achieve spontaneous pregnancy in one year of regular unprotected intercourse (1). Epidemiological studies have shown the acute decline in semen quality and human male fertility with underlying geographic variation in prevalence. On a global scale, the disease affects approximately 15% to 20% of married couples (2), of which male-factor contributes 50% of cases (3) and about 60-75% of male infertility cases are idiopathic (4). The prevalent rate in the UK and USA is estimated to be 6% and 10% respectively (5), with the highest rates in Africa and Central/Eastern Europe (6). However, the infertility rate in Nigeria may attract more attention as some studies have reported worrisome 65% and 35% prevalence rates for primary and secondary infertility, respectively (7).

There is growing reproductive health concern about the considerable decrease in sperm quality and fertility. Although the cause of male infertility is understood in some cases of underlying pathological conditions, most cases are due to poor semen quality or sperm abnormalities of unknown causes (2). In recent years, there has been an increasing interest in the contribution of occupational and environmental exposures to toxic pollutants to declining sperm concentrations and human male fertility (8). Evidence suggests that exposure to toxic heavy metals such as Pb and Cd may cause impairments in sperm count, motility and morphology (9), although the role of Pb and Cd on hormone concentrations, male fertility and sperm parameters, reports have been limited and equivocal in the published papers (8, 10, 11). However, levels of Pb and Cd in seminal fluid have been demonstrated for abnormal spermatozoa function and fertility (5, 12). Low to moderate Pb exposure has been associated with aberrations in sperm concentration, motility, viability and morphology (13). Studies conducted in non-African populations showed significant negative effects of Pb or Cd exposure levels on semen quality (14-18). 

The cause for the over 12 million infertile men and women in Nigeria is largely unknown (19). Although there is a general belief that the most common cause of infertility in Nigeria is infection (20), but there are cases of treatment of infections that failed to reverse infertility (19). Nigerian environments have been reported to be highly polluted with toxic metals, especially Pb and Cd (20). There exist many sources of environmental Pb exposure in Nigeria. At the top of the list is leaded gasoline with average Pb content of 0.66 g /L currently in use (20), others include burning of electronic wastes in open air with crude waste management practices and consumption of uncertified herbal remedies reported to contain high levels of Pb and Cd (21). However, there are higher rates of irreversible oligo- or azoospermia than most other causes of infertility among men in Nigeria (20). Studies have shown that seminal plasma level of toxic metals is a more reliable index to reveal exposure, accumulation and effect on reproductive health (22). However, there is paucity of data on the possible effect of Pb and Cd on fertility. Therefore, the objective of this study was to determine the levels of Pb and Cd in blood and seminal plasma in non-occupationally exposed infertile Nigerian men in Ebonyi State, which is known to have large deposits of heavy metals and correlate their levels with semen quality.

## Materials and methods

***Recruitment of subjects:*** This cross-sectional study investigated men aged 20–45 years. Seventy-five (75) male partners of women consulting for infertility at the Fertility Clinic Unit, Department of Obstetrics and Gynecology of Our Lady’s Hospital, Abakaliki, South-East Nigeria between July and November, 2014 were recruited for enrolment in this study. The men with primary and secondary infertility were referred for semen analysis. Sociodemographic data and obstetric history were obtained by structured questionnaire. Body mass index (BMI) for each participant was calculated based on height and weight. 

***Inclusion criteria:*** We included males of couples living together with regular unprotected coitus for at least a period of ≥ 1 year without conception.

***Exclusion criteria:*** Medical examination of potential participants was performed. Males with genital infection, use of contraceptives, and testicularvaricocele, and smoking/chronic alcohol intake, micronutrient supplementation for the last three months, surgery, and chronic illness including endocrine diseases were excluded from the study. Men currently on fertility drugs and those that are occupationally exposed to toxic metals were also excluded.

***Study consent/Ethical approval:*** A written consent of each participant that merited our selection criteria was taken after explaining the aim and objectives of the study and its benefits on individual and society. Also, the Ethics and Research Committees of the Federal Teaching Hospital, Ebonyi State University, Abakaliki approved the study protocols.

***Collection of Blood and Semen:*** Semen samples were obtained in private by masturbation into a sterile wide-mouth and metal-free glass container after 3 days of sexual abstinence. Collection of spilled semen and prior use of antibiotics were avoided. Fasting venous blood (5.0 ml) was also collected from each participant into heavy metal-free lithium-heparin sample bottle for determination of lead and cadmium. The samples were centrifuged at 2000 g for 5 minutes after which plasma was isolated and stored frozen (-20^O^C) until analysis.

***Semen parameter analysis:*** Standard semen parameters (volume, concentration, motility, morphology) were immediately evaluated according to guidelines of the WHO Laboratory Manual for the Examination of Human Semen (23), after liquefaction at 37°C for 30 min and within 1 h of semen collection. The sample analysis was done by the same Medical Laboratory Scientists of the Fertility Clinic Unit, Department of Obstetrics and Gynaecology of Our Lady’s Hospital, Abakaliki, South-East Nigeria, to avoid inter-laboratory and inter-individual variation. Quality control measures were adhered to throughout the study. Each sample was analysed twice, successively. After semen analysis, selected participants were divided into 3 categories using the normal sperm concentration of > 20 × 10^6^ sperm cell/ml (23). 

***Lead and cadmium analysis:*** Semen samples collected for the analyses of semen quality were also used to determine Pb and Cd levels. The semen was spurn at 15000 g for 15minutes. The supernatant was separated and used as seminal plasma, frozen at -20^o^C until analyses for lead and cadmium determination. Flame atomic absorption spectrophotometer (Buck Scientific, AVG 210) was used to determine the concentrations of lead and cadmium in seminal and blood plasma.

***Statistical analysis:*** The data obtained were statistically evaluated using statistical package for Social Science Program (SPSS^®^) for Windows^®^ version 20.0. Values are represented as mean ± standard deviation for all variables. One-way analysis of variance (one-way ANOVA) was used to compare the means while relationships between parameters were determined using Pearson correlation analysis. Values were considered statistically significant at p value <0.05.

## Results

***Demographic characteristics:*** Seventy-three (73) males conforming to the inclusion criteria were recruited in the study. All participants were married and infertile couples without occupational exposure to Pb and Cd. Based on sperm concentration, we found normospermia (36 men, control), oligospermia (22 men) and azoospermia (15 men). The males ranged in age from 20 to 45 years with a mean age of 37.1 (SD = 7.0 yrs). As shown in Table 1, the mean of age of the normospermics (36.1 ± 7.0 yrs) was lower compared to azoospermics (39.5 ± 7.3 yrs) and oligospermics (37.3 ± 6.9 yrs), but the difference was not significant (p = 0.252). Also, the BMI of the three groups were comparable (p = 0.141).

***Seminal parameters:*** From table 1, the mean semen volume of the participants was comparable. However, mean sperm count in normospermics (117.97 × 10^6^ cell/ml) was significantly higher (p = 0.00) than the azoospermics (0.00 ×10^6 ^cell/ml) and oligospermics (10.86 × 10^6^ cell/ml), whereas the percentage total motility and morphology were not significantly different except in azoospermics.

***Cadmium and lead levels in blood and seminal plasma:*** From table 2, seminal and blood plasma cadmium as well as blood plasma leadwere significantly (p < 0.01) higher in azospermic and oligospermic men in comparison to normospermic men, whereas values in azospermic and oligospermic men were comparable (p > 0.05). However, while seminal plasma lead was significantly (p < 0.01) higher in oligospermic and normospernic men than in azospermic men, it was comparable between oligospermic and normospermic men. Again, while significant inverse associations (p < 0.01) was found between blood and seminal cadmium levels and sperm count, motility and morphology, blood lead was inversely correlated with sperm count only.

***Correlation of lead and cadmium with seminal parameters:*** Table 3 shows the Pearson correlation analysis of blood and seminal plasma cadmium with seminal parameters. There were significant (p < 0.01) inverse correlations of blood plasma Cd with sperm count, sperm motility, and sperm morphology. Similarly, we observed that seminal cadmium was inversely correlated with sperm count, sperm motility, and sperm morphology. 

**Table 1:** Demographic characteristics and seminal parameters of azoospaemic, oligospaemic and normospaemic men

**Parameter**	**Azoospaemic (n = 15)**	**Oligospaemic (n = 22)**	**Normospaemic (n = 36)**
Age (years)	39.54 ± 7.30	37.32 ± 6.90	36.08 ± 6.96
BMI (kg/m^2^)	28.4 ± 3.60	27.99 ± 4.63	26.62 ± 5.36
Volume (ml)	2.2 ± 0.99	2.91 ± 1.38	2.92 ± 1.32
Count (×10^6^cell/ml)	0.00 ± 0.00^c^	10.86 ± 3.11^b^	117.97 ± 48.55^a^
Total Motility (%)	0.00 ± 0.00^c^	44.32 ± 16.78^b^	55.83 ± 16.63^a^
Morphology (%)	0.00 ± 0.00^c^	46.82 ± 9.46^b^	58.33 ± 13.63^a^

Values are expressed as mean ± SD. Different superscripts along the same row are significantly different (p < 0.05).

**Table 2:** Seminal and blood plasma cadmium and lead levels in azoospaemic, oligospaemic and normospaemic men (μg/dl)

**Parameter **	**Azoospaemic (n = 15)**	**Oligospaemic (n = 22)**	**Normospaemic (n = 36)**
Blood plasma cadmium	0.98 ± 0.49^a^	0.77 ± 0.36^a^	0.43 ± 0.29^b^
Seminal plasma cadmium	0.89 ± 0.41^a^	1.14 ± 0.43^a^	0.49 ± 0.31^b^
Blood plasma lead	1.71 ± 0.98^a^	2.05 ± 0.97^a^	1.49 ± 0.59^b^
Seminal plasma lead	0.44 ± 0.49^a^	1.22 ± 0.81^b^	0.85 ± 0.86^b^

Values are expressed as mean ± SD. Different superscripts along the same row are significantly different (p < 0.05).

However, in table 4, plasma lead seemed to correlate inversely with all the sperm parameters only its correlation with sperm count was statistically significant (p = 0.02). However, seminal lead demonstrates non-significant positive correlation with semen volume, sperm motility, and sperm morphology.

***Correlation of lead and cadmium with seminal parameters:*** Table 3 shows the Pearson correlation analysis of blood and seminal plasma cadmium with seminal parameters. There were significant (p < 0.01) inverse correlations of blood plasma Cd with sperm count, sperm motility, and sperm morphology. Similarly, we observed that seminal cadmium was inversely correlated with sperm count, sperm motility, and sperm morphology. However, in table 4, plasma lead seemed to correlate inversely with all the sperm parameters only its correlation with sperm count was statistically significant (p = 0.02). However, seminal lead demonstrates non-significant positive correlation with semen volume, sperm motility, and sperm morphology.

## Discussion

Trends in male reproductive health have been reported for acute decline in semen quality and fertility, which have been associated with occupational and environmental chemical exposure based on animal and human studies (24). Although there are inconsistent reports of association of Pb and Cd with the decline in semen quality (25), it is plausible that the several environmental factors may cause male infertility, particularly that the adverse effects of toxic heavy metals on humans is well documented (10). However, it is consistent in the literature that male infertility is variable, with a multitude of influencing geographical differences, including environmental and lifestyle factors (2, 10). 

This study investigated association of blood and seminal plasma Cd and Pb concentrations with semen quality in non-occupationally exposed infertile Nigerian men undergoing infertility evaluation. The overall results of this study indicate that even considerably low levels of blood and seminal plasma Pb (0.44–2.05 μg/dl) and Cd (0.43–1.14 μg/dl) might reduce the human semen quality. We found that Cd and Pb levels in azoospermic and oligospermic blood and seminal plasma were significantly higher compared to normospermic men (Table 2). This may be associated with adverse reduction in the basic semen parameters: sperm concentration, motility, and morphology observed in the current study (Table 1). Our results are in consonance with the previous evidence that indicate deleterious effects of Pb and Cd on the reproductive system of Nigerian men (12, 22) and other populations across the globe (13-15). Further, the association of Pb with abnormal sperm concentration in the study supports the recent findings of Wu et al. (26) that semen Pb concentration was significantly higher among the patients with lower sperm count. Benoff et al. (17) and Mendiola et al. (18) reported that cadmium concentration at the level of 0.028 or 0.08 μg/dl in seminal plasma, respectively, might affect one or more seminal parameters. On the contrary however, the reports of Hovatta et al. (27) and Xu et al. (28) suggested that there are no association between seminal lead and cadmium concentrations and sperm concentration, motility and morphology.

**Table 3:** Pearson correlation of seminal and plasma cadmium levels with semen parameters in infertile men

**Parameters**	**Plasma cadmium**		**Seminal cadmium**	
	**R value**	**P** **value**	**R value**	**P** **value**
Semen volume (ml)	-0.147	0.22	-0.095	0.43
Sperm count (×10^6 ^cell/ml)	-0.455	0.001*	-0.476	0.001*
Total Motility (%)	-0.385	0.001*	-0.249	0.032*
Morphology (%)	-0.413	0.001*	-0.304	0.01*

* Statistically significant correlation at p < 0.05

**Table 4:** Pearson correlation of seminal and plasma lead levels with semen parameters in infertile men

**Parameters **	**Plasma lead**		**Seminal lead**	
	**R value**	**P** **value**	**R value**	**P** **value**
Semen volume (ml)	-0.132	0.27	0.009	0.94
Sperm count (×10^6 ^cell/ml)	-0.280	0.02*	-0.06	0.62
Total Motility (%)	-0.092	0.44	0.134	0.27
Morphology (%)	-0.081	0.50	0.177	0.14

* Statistically significant correlation at p < 0.05

Omu et al. reported non-significant association of Cd with semen parameters in normozoospermic, oligozoospermic and azoospermic men (29). Reports of other investigators (30) also suggest that no correlation was found between semen Cd level of 0.04 μg/dl and sperm parameters, although our findings from correlation analysis are inconsistent with this report. In this study, statistically significant inverse correlation (p < 0.01) was observed between Cd and sperm count, total motility and morphology (Table 3). However, Pb level was only significantly negatively correlated with sperm count (Table 4). By implication therefore, Cd may demonstrate higher reproductive toxicity than Pb in non-occupationally exposed men. Moreover, Saksena et al. had suggested that epididymis and seminal vesicles contains the highest concentration of Cd in the body and appears to have a high affinity for the male reproductive system (31). Human spermatozoa are reservoir of antioxidant defense system, including zinc (32). Cadmium and lead are well-known environmental toxicants that induce oxidative stress due to ROS accumulation (33). Since the metabolism of Cd is closely related to Zn metabolism, the potential for Cd to reduce Zn level and generate reactive oxygen species may be exacerbated, leading to alterations in the antioxidant defense system and imposing oxidative stress and lipid peroxidation on spermatozoa. The oxidative impact of Cd on spermatogenesis and sperm count might be associated with their deleterious effect on testicular structure and function as shown in previous studies (33). This indicates the important role of Cd in male reproduction. It is then possible that environmental exposure to Cd through e-wastes, cigarette smoke, alcoholic beverages, municipal refuse, automobile battery discharge in highly polluted environments like Nigeria (8, 20) may contribute to Cd accumulation in male reproductive tissues to cause infertility. This may, in part, explains the substantial contribution of male-factor to infertility (3), and in Nigeria the worrisome increase in prevalence rates (7).

Existing reports on the role of Pb in male infertility is still unclear and available papers are controversial (8, 26, 34). The previous reports suggest that environmental exposures to Pb do not significantly contribute to men infertility (8). Although, the blood and seminal plasma Pb in this study failed to show marked correlation with seminal parameters as in previous studies (34, 35) the Pb level profoundly reduced the sperm count in oligospermic men. Animal studies have reported that Pb affects spermatogenesis and reduces number of spermatozoa within the epididymis in mice administered lead (36), and resists spermatogenesis in rats (37); however similar data in humans is generally limited. Lead-detrimental spermatogenesis rather than lead-altered hypothalamic-pituitary-gonadal function may be responsible for the association between seminal lead concentration and sperm count (26, 38). In an experimental animal study, lead has been shown to contribute to alterations in sperm chromatin condensation which has been associated with a low percentage of fertilization (35, 39). The effect of Pb on sperm nucleus in the epididymis by binding to nuclear sulfhydryl groups from the DNA–protamine complex in the epididymis delays nuclear decondensation (33). Increased seminal plasma Pb concentration may exhibit adverse effect on spermatogenesis, probably expressed as infertility among Nigerian men.

***Conclusion:*** We found some significant relationships between Pb and Cd and semen quality in the present study. Our results suggest that environmental exposure to Pb and Cd may adversely affects fertility in men resident in Abakaliki, Southeastern Nigeria.
